# The Preferred Locations of Meningioma According to Different Biological Characteristics Based on Voxel-Wise Analysis

**DOI:** 10.3389/fonc.2020.01412

**Published:** 2020-08-21

**Authors:** Chongran Sun, Zhangqi Dou, Jiawei Wu, Biao Jiang, Yasaman Iranmanesh, Xiaobo Yu, Jianru Li, Hang Zhou, Chen Zhong, Yucong Peng, Jianfeng Zhuang, Qian Yu, Xinyan Wu, Feng Yan, Qi Xie, Gao Chen

**Affiliations:** ^1^Department of Neurosurgery, Second Affiliated Hospital, School of Medicine, Zhejiang University, Hangzhou, China; ^2^Department of Radiology, Second Affiliated Hospital, School of Medicine, Zhejiang University, Hangzhou, China; ^3^School of Life Science, Westlake University, Hangzhou, China

**Keywords:** meningiomas, magnetic resonance imaging, voxel-wise analysis, lesion location preference, recurrence

## Abstract

**Objective:** Meningiomas presented preferred intracranial distribution, which may reflect potential biological natures. This study aimed to analyze the preferred locations of meningioma according to different biological characteristics.

**Method:** A total of 1,107 patients pathologically diagnosed with meningiomas between January 2012 and December 2016 were retrospectively analyzed. Preoperative MRI were normalized, and lesions were semiautomatically segmented. The stereospecific frequency and p value heatmaps were constructed to compare two biological phenotypes using two-tailed Fisher's exact test. Age, sex, WHO grades, extent of resection (EOR), recurrence, and immunohistochemical markers including p53, Ki67, epithelial membrane antigen (EMA), progesterone receptor (PR), and CD34 were statistically analyzed. Recurrence-free survival (RFS) were analyzed by Kaplan–Meier method.

**Result:** Of 1,107 cases, convexity (20.8%), parasagittal (16.1%), and falx (11.4%) were the most predominant loci of meningiomas. The *p*-value heatmap suggested lesion predominance in the left frontal and occipital convexity among older patients while in the left sphenoid wing, and right falx, parasellar/cavernous sinus, and middle fossa among younger patients. Lesions located at anterior fossa and frontal structures were more frequently seen in the male while left parietal falx and tentorial regions, and right cerebellopontine angle in the female. Grades II and III lesions presented predominance in the frontal structures compared with grade I ones. Meningiomas at the left parasagittal sinus and falx, tentorium, intraventricular regions, and skull-base structures were significantly to receive subtotal resection. Lesions with p53 positivity were statistically located at the left frontal regions and parasellar/cavernous sinus, higher Ki67 index at the left frontal and bilateral parietal convexity and right parasellar/cavernous sinus, EMA negativity at the right olfactory groove and left middle fossa, and CD34 positivity at the sellar regions and right sphenoid wing. Tumor recurrence rates for grades I, II, and III were 2.8, 7.9, and 53.8%, respectively. Inferior RFS, higher Ki67 index, grades II and III, and a larger preoperative volume were observed in older patients. Recurrent meningiomas were more frequently found at the occipital convexity, tentorium, sellar regions, parasagittal sinus, and left sphenoid wing.

**Conclusion:** The preferred locations of meningioma could be observed according to different biological characteristics, which might be helpful for clinical decisions.

## Introduction

Meningiomas account for 37.6% of all primary central nervous system (CNS) tumors and 53.3% of non-malignant intracranial tumors, with an incidence rate of 8.6 per 100,000 (1). Meningiomas originate from the arachnoidal cap cell and are histologically divided into grades I, II (atypical), and III (anaplastic), according to the 2016 WHO classification (2, 3). Approximately 80% of the meningiomas are grade I with benign behaviors, while the high-grade lesions (grades II and III) tend to recur and metastasize (4). Surgery is recognized as the first option for treating patients with meningioma, pursuing the primary goal of complete resection (Simpson grade I) (5). Patients with lesions in favorable locations (e.g., convexity meningiomas) presented improved recurrence-free survival (RFS) by extensive resection (6). Besides surgery, however, radiotherapy or radiosurgery is required for meningiomas in uneasily accessible locations (5). Moreover, the meningioma locations are related to the symptoms, tumor histology, and the prognostic value of Simpson classification. Skull-base lesions insult memory more seriously than convexity ones do (7). Atypical meningiomas are associated with a location on the convexity, and Simpson grade is correlated to the high risk of recurrence for tumors in this location instead of falx and posterior fossa (8–10). Therefore, the location-specific difference in meningioma greatly influences clinical decisions and therapeutic strategies.

The spatial distribution of meningioma has long been investigated with clinical interest to explore the location-specific difference. A study in the 1930s suggested that the anterior one-third of the superior sagittal sinus was commonly affected (11). Moreover, this result was verified by Hirayama et al. using voxel-based lesion mapping for 260 meningiomas (12). The authors also discovered more frequent distribution in skull-base structures and regions around central sulcus and the sylvian fissure. Given this, the voxel-wise analysis based on MRI is a valuable method to show the spatial landscape of brain tumors, which have been applied in glioblastoma, brain metastases, and primary CNS lymphoma (13–16). Notably, the preferred locations of glioblastoma were statistically compared and visualized by atlas in terms of biological features and genetic alterations (13, 14). Similarly, biological and clinical characteristics are of great importance for meningiomas and might be associated with the location. A study indicated that WHO grades, Ki67-MIB1, and progesterone receptor (PR) expression differed depending on tumor locations (17).

The present study, therefore, used a large surgery-treated patient cohort at our institution and applied voxel-wise mapping and Fisher's exact test to visualize the preferred locations of meningioma according to different biological characteristics. Location analysis might improve the clinical understanding of meningiomas.

## Materials and Methods

### Patient Cohort

Patients with meningiomas who received surgery between January 2012 and December 2016 at our institution were reviewed. Preoperative contrast-enhanced T1-weighted MRI (CE-T1WI) and histopathological reports were consecutively extracted from the institutional medical database. A total of 1,107 patients were included. The extent of resection (EOR), including gross total resection (GTR, Simpson grades I and II) and subtotal resection (Simpson grades III and IV), was classified according to surgical records and the recheck of postoperative MRI, which were reviewed by a senior neuroradiologist (BJ). The biological characteristics include age, sex, WHO grade, EOR, recurrence, and the expression of p53, Ki67, epithelial membrane antigen (EMA), PR, and CD34.

### Patient Consent

The inclusion process was approved by the institutional ethical committee on human clinical research. A general informed consent agreement, stating that the clinical, pathological, and imaging data with privacy protection might be used for teaching and scientific research, was signed by every patient as soon as hospitalized. Because of the retrospective nature of the current study with no clinical intervention, the specific informed consent agreement to a project was waived by the ethical committee. Medical records were desensitized for privacy protection.

### Magnetic Resonance Imaging

The patients underwent an either 1.5-(Signa Excite, GE Healthcare, Milwaukee, Wisconsin) or 3.0-T (Discovery 750, GE Healthcare, Milwaukee, Wisconsin) MRI. Intravenous injection of gadodiamide (0.2 ml/kg body weight, up to a maximum of 20 ml, Omniscan, GE Healthcare) was used to obtain the CE-T1WI. The last scan before surgery demonstrating meningioma was used for analyses.

### Definition of Tumor Location

The locations of meningioma were identified according to the surgical description and the dura mater attached with a tumor in imaging. Locations were classified as the previous study with modifications (12). These included convexity, parasagittal sinus, falx, tentorium, cerebellar convexity, cerebellopontine angle, sphenoid wing, parasellar/cavernous sinus, tuberculum sellae/planum sphenoidale/anterior clinoid process, middle fossa, olfactory groove, clival–petroclival, foramen magnum, intraventricular, and other types (multiple/orbital/jugular foramen). A neurosurgeon (CS) and a neuroradiologist (BJ) reviewed the results.

### Image Normalization and Segmentation

Images were exported in the standard Digital Imaging and Communications in Medicine (DICOM) format. They then were converted into the Neuroimaging Informatics Technology Initiative (NIfTI) format using dcm2nii converter software (University of Nottingham School of Psychology, Nottingham, UK). The axial images were selected. Statistical Parametric Mapping Software version 12 (SPM12, Institute of Neurology, University College London, London, UK) in MATLAB (version R2012a, The MathWorks, Natick, MA, USA) was used to register the images to a standard brain template (MNI152; Montreal Neurological Institute, McGill University, Montreal, Quebec, Canada) for normalization (15, 16). The regions of interest (ROIs) in normalized images were semiautomatically segmented using 3D Slicer (version 4.10.0; http://www.slicer.org/) and its “Grow from Seed” module (18). The segmentation was performed by two neurosurgeons and reviewed by another neurosurgeon and the neuroradiologist.

### Stereospecific Frequency and p-value Heatmaps

MRIcron (University of Nottingham School of Psychology, Nottingham, UK) was used to superimpose the ROIs on MNI152 to construct stereospecific frequency heatmaps. The p value heatmaps were created to compare two different phenotypes under one characteristic (e.g., comparing old with young patients) and calculate the significance of a voxel. The two-tailed Fisher's exact test was performed with custom Python scripts, as previously described by Ellingson et al. (13, 14).

$$\begin{array}{l} p=\frac{\left(a+b\right)!\left(c+d\right)!\left(a+c\right)!\left(b+d\right)!}{a!b!c!d!n!} \end{array}$$

In the formula, “*a*” is the frequency of tumor occurrence under phenotype A, “*b*” is the frequency of tumor occurrence under phenotype B, “*c*” is the frequency of tumor-free patients under phenotype A, “*d*” is the frequency of tumor-free patients under phenotype B, and “*n*” is the total number of patients.

### Statistical Analyses

The normalized tumor volume was calculated by multiplying the number of voxels within the ROI by the volume of a single voxel (0.08 mm^3^) in MNI152, approximating to the lesion volume before normalization. Recurrence-free survival (RFS) was determined by the Kaplan–Meier analysis with the log-rank test. The Kruskal–Wallis and Dunn's multiple comparison test were used when appropriate, and data were presented as mean ± standard error of the mean (SEM). GraphPad Prism (version 8.0.2; GraphPad Software, San Diego, CA, USA) and SPSS (version 22.0; IBM SPSS Statistics, Armonk, NY, USA) were used for all statistical analyses. *p* < 0.05 was deemed significant.

## Results

### Demographics

Among the 1,107 patients, the median age was 56 years, and the ratio of male to female was 3:7. According to the WHO classification, 993 (89.7%), 101 (9.1%), and 13 (1.2%) were grades I, II, and III, respectively. WHO grade I consisted of 717 female (72.2%) and 276 male (27.8%). WHO grades II and III consisted of 68 female (59.6%) and 46 male (40.4%). The gender distribution according to different WHO grades was significantly different (Supplementary Figure 1, *p* = 0.005). Convexity (20.8%), parasagittal (16.1%), and falx (11.4%) were the three most common locations affected by meningiomas, followed by skull-base structures including sphenoid wing (9.8%), cerebellopontine angle (CPA, 7.7%), tuberculum sellae/planum sphenoidale/anterior clinoid process (7.6%), and olfactory groove (6.0%). Ninety percent of the patients received GTR (Simpson grades I and II). Forty-three patients had recurrence during the follow-up visits, and the recurrence rates for grades I, II, and III were 2.8, 7.9, and 53.8%. The demographics are summarized in Table 1.

**Table 1:** Demographics of 1,107 patients with meningiomas.

**Characteristics**	**Number (%)**
**Age**	
Range	13–85
Median	56
**Sex**	
Male	322 (29.1)
Female	785 (70.9)
**WHO grades**	
I	993 (89.7)
II	101 (9.1)
III	13 (1.2)
**Locations**	
Convexity	230 (20.8)
Parasagittal sinus	179 (16.1)
Falx	126 (11.4)
Tentorium	94 (8.5)
Cerebellar convexity	10 (0.9)
Cerebellopontine angle	86 (7.7)
Sphenoid wing	109 (9.8)
Parasellar/cavernous sinus	32 (2.9)
Tuberculum sellae/planum sphenoidale/anterior clinoid process	84 (7.6)
Middle fossa	14 (1.3)
Olfactory groove	66 (6.0)
Clival-petroclival	21 (1.9)
Foramen magnum	3 (0.3)
Intraventricular	22 (2.0)
Others (multiple/orbital/jugular foramen)	31 (2.8)
Extent of resection^*^	
Gross total resection	945 (90.0)
Subtotal resection	105 (10.0)
**Recurrence rates^#^**	
I	28 (2.8)
II	8 (7.9)
III	7 (53.8)

* *The results of EOR were lost in 57 patients; thus, the EOR was evaluated in 1,050 patients*.

# *The recurrence rates were calculated based on the number of patients of the corresponding WHO grade*.

### Tumor Volume

**Figure 1:**
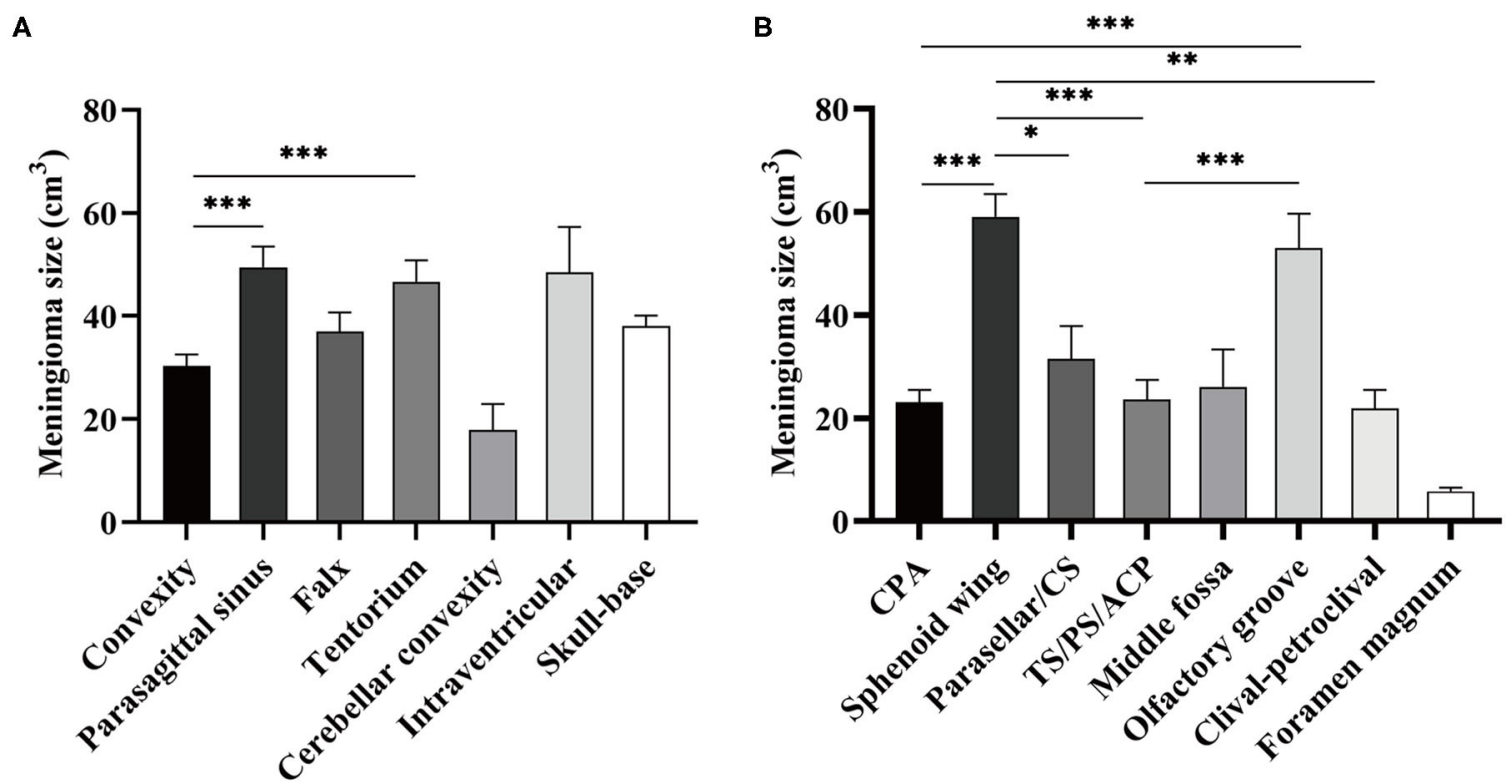
Comparison of normalized tumor volume according to the locations of meningioma. **(A)** Meningioma size was compared between skull- and non-skull-base locations. **(B)** Meningioma size was compared among eight skull-base locations. CS refers to cavernous sinus; TS/PS/ACP refers to tuberculum sellae/planum sphenoidale/anterior clinoid process. ^*^p < 0.05, ^**^p < 0.01, and ^***^p < 0.001, respectively.

The median preoperative tumor volume after normalization was 22.828 cm^3^ (95% confidence interval, 20.64–25.37). Although no significance of tumor volume was found comparing non-skull-base with skull-base meningiomas, convexity meningiomas were statistically smaller than the parasagittal and tentorial ones (Figure 1A). Further comparison among skull-base lesions demonstrated a larger volume of the sphenoid wing and olfactory groove meningiomas than lesions at other locations (Figure 1B).

### Stereospecific Frequency Heatmap

**Figure 2:**
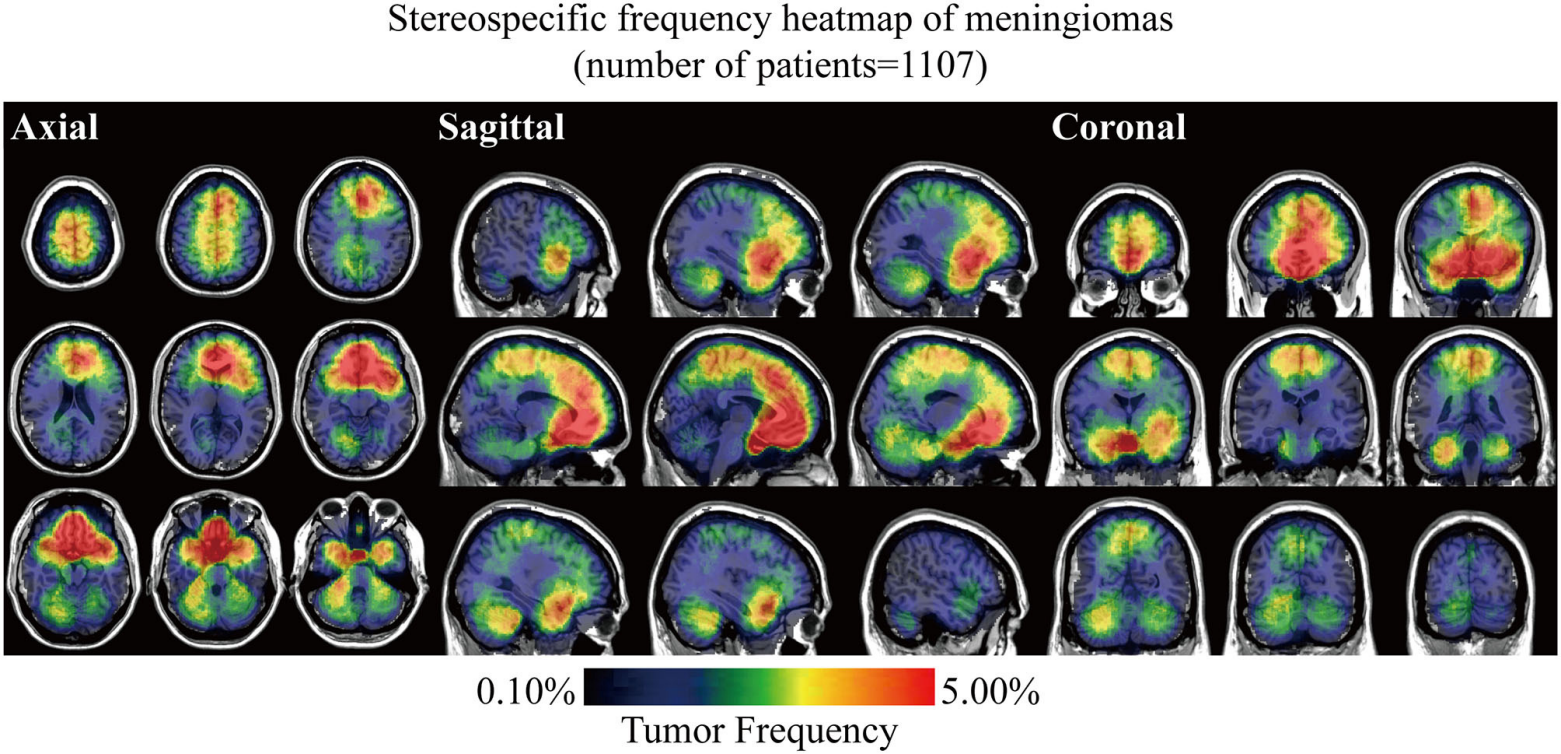
Stereospecific frequency heatmap of meningiomas with axial, sagittal, and coronal positions. Lesions in 1,107 patients were normalized, segmented, and superimposed on the MNI152. The color ranging from dark blue to red indicated the tumor frequency from 0 to 5% and above.

Stereospecific frequency heatmap was constructed by ROIs overlapping to visualize the spatial landscape of meningiomas. The color ranging from dark blue to red indicated the tumor frequency from 0 to 5% and above (Figure 2). The results indicated that lesions preferred to distribute at the anterior two-thirds of the superior sagittal sinus and falx, olfactory groove, tuberculum sellae/planum sphenoidale/anterior clinoid process, parasellar/cavernous sinus, and CPA. Although convexity meningiomas accounted for the largest proportion of all the cases, no particularly densely distributed area was observed in the convexity. Mild left lateralization was found. The laterality was further analyzed according to locations and biological characteristics, including age, sex, and WHO grade, but no significance was found (Supplementary Figure 2 and Supplementary Table 1).

### p-value Heatmaps

**Figure 3:**
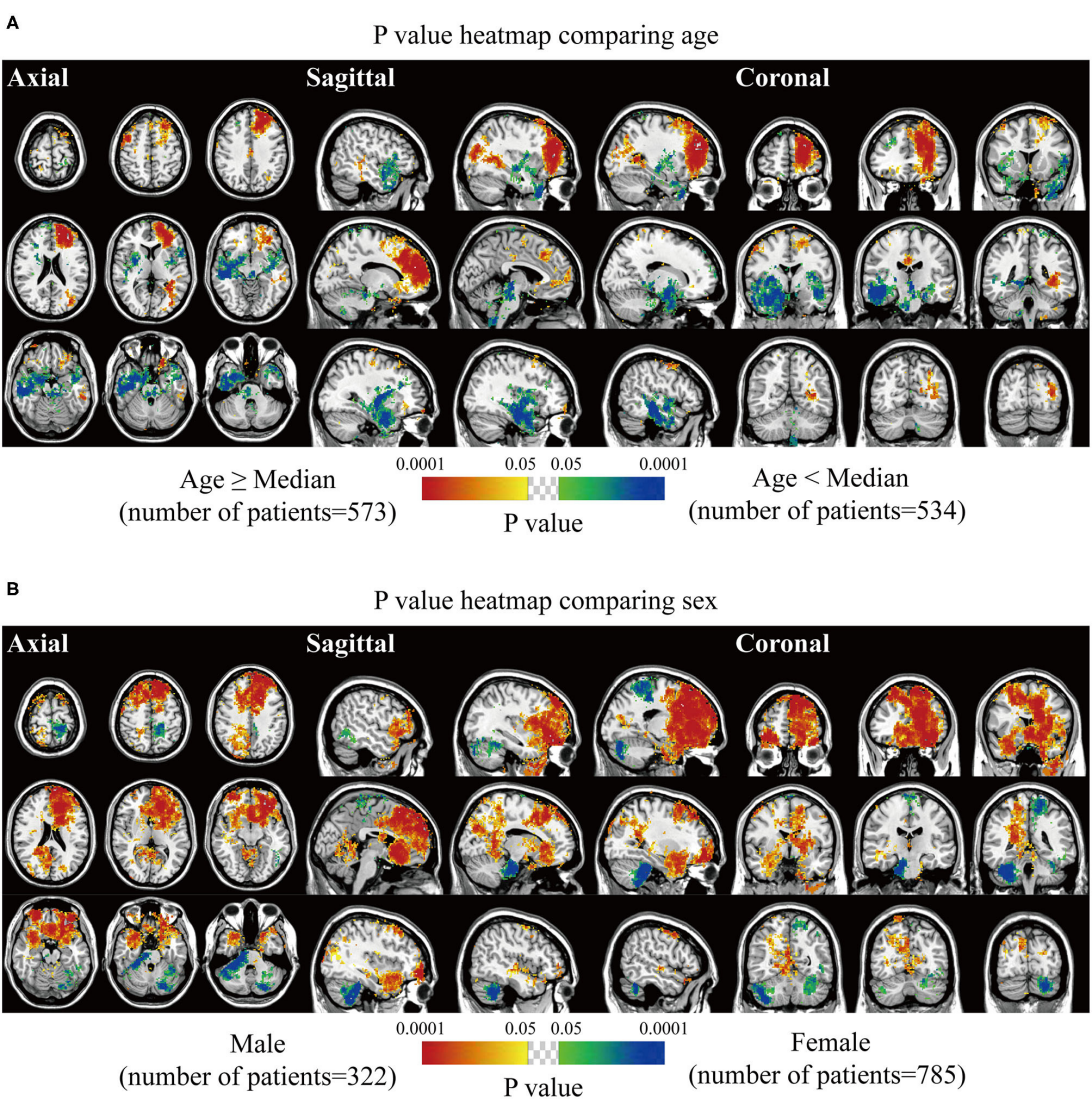
*p*-value heatmaps of meningioma comparing age and sex. **(A)** *p*-value heatmap showed statistically significant clusters after comparing older (age ≥ 56 years) with younger (age < 56 years) patients. **(B)** *p*-value heatmap showed statistically significant clusters after comparing male with female patients. The color ranging from green to dark blue, and bright yellow to red, both indicated the *p*-value from 0.05 to 0.0001.

**Figure 4:**
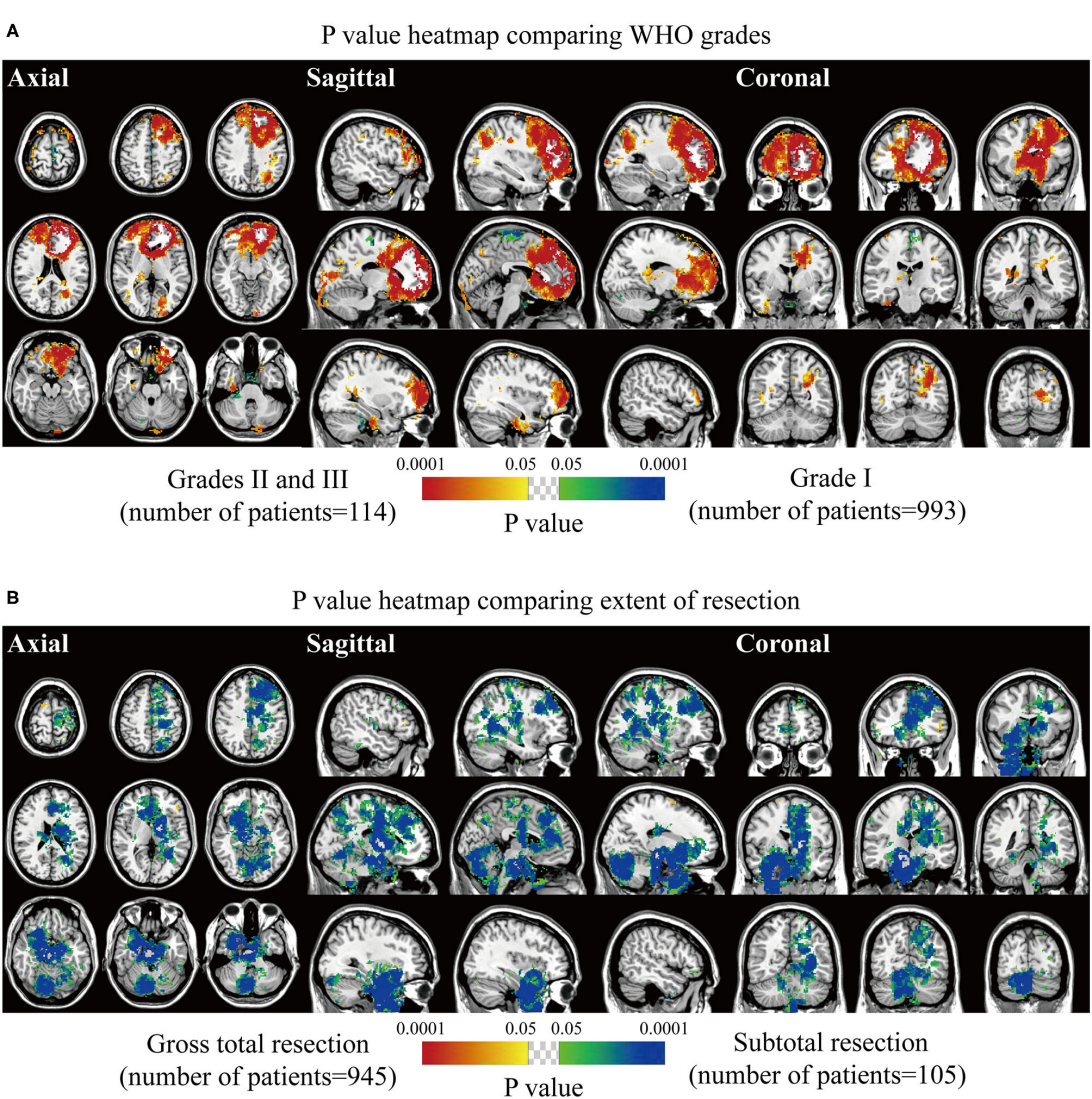
*p*-value heatmaps of meningioma comparing WHO grade and extent of resection. **(A)** *p*-value heatmap showed statistically significant clusters after comparing grade I lesions with grades II and III ones. **(B)** *p*-value heatmap showed statistically significant clusters after comparing patients received gross total resection with ones received subtotal resection. The color ranging from green to dark blue, and bright yellow to red, both indicated the *p*-value from 0.05 to 0.0001.

Biological characteristics including age, sex, WHO grade, EOR, and expression of p53, Ki67, EMA, PR, and CD34 were statistically analyzed. Two different phenotypes under one characteristic were compared, and significant voxels were visualized to explore the preferred locations. The median age of 56 years was set as the cutoff value to stratify the patients. Results suggested statistically significant clusters in the left frontal and occipital convexity in older patients while in the skull-base structures including sphenoid wing, and right falx, parasellar/cavernous sinus and middle fossa in younger patients (Figure 3A). Tumor frequency results showed the predominance of the anterior fossa, frontal structures, and tentorial regions in the male sex compared to female sex with the predominance of the left parietal falx, tentorium, and cerebellar convexity, and right CPA (Figure 3B). *p*-value heatmap based on the WHO classification identified clusters in the frontal structures and left parietal and occipital convexity as more frequently associated with high-grade meningiomas (grades II and III) and clusters in the left parasagittal sinus, right CPA, and sellar regions as more frequently associated with grade I meningiomas (Figure 4A). Significant clusters in the left parasagittal sinus and falx, tentorium, intraventricular regions, and skull-base structures (e.g., sellar regions and sphenoid wing) were identified as more frequently associated with meningiomas received subtotal resection (Figure 4B).

**Figure 5:**
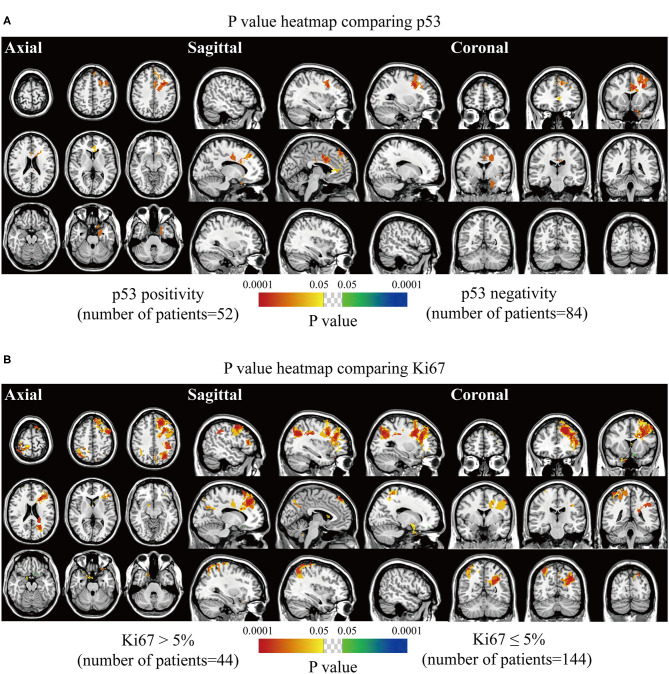
*p*-value heatmaps of meningioma comparing immunohistochemical p53 and Ki67. **(A)** *p*-value heatmap showed statistically significant clusters after comparing p53 positivity with negativity. **(B)** *p*-value heatmap showed statistically significant clusters after comparing Ki67 > 5% lesions with Ki67 ≤ 5% ones. The color ranging from green to dark blue, and bright yellow to red, both indicated the p value from 0.05 to 0.0001.

**Figure 6:**
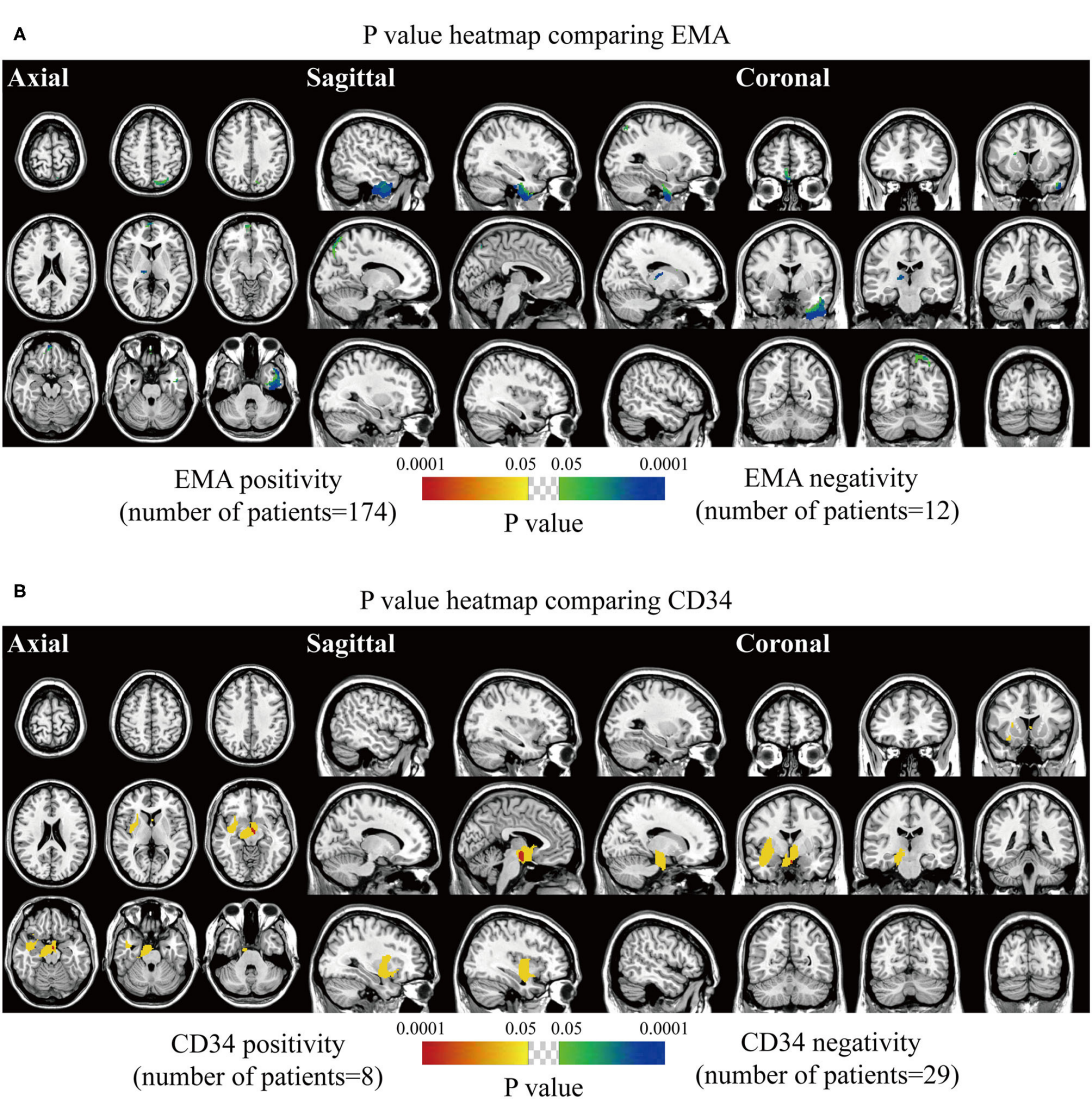
*p*-value heatmaps of meningioma comparing immunohistochemical epithelial membrane antigen (EMA) and CD34. **(A)** *p*-value heatmap showed statistically significant clusters after comparing EMA positivity with negativity. **(B)** *p*-value heatmap showed statistically significant clusters after comparing CD34 positivity with negativity. The color ranging from green to dark blue, and bright yellow to red, both indicated the *p*-value from 0.05 to 0.0001.

The spatially distinct regions in the left falx and parasellar/cavernous sinus occurred at a significantly higher frequency in lesions with positive expression of p53 (Figure 5A). The predominance in the left frontal and bilateral parietal convexity and right parasellar/cavernous sinus was significantly associated with Ki67 > 5% (Figure 5B). Lesions with negative expression of EMA frequently occurred in the right olfactory groove and left middle fossa (Figure 6A). Tumor frequency results showed the predominance of sellar regions and right sphenoid wing in lesions with positive expression of CD34 (Figure 6B). No significant location was found for PR.

### Tumor Recurrence

**Figure 7:**
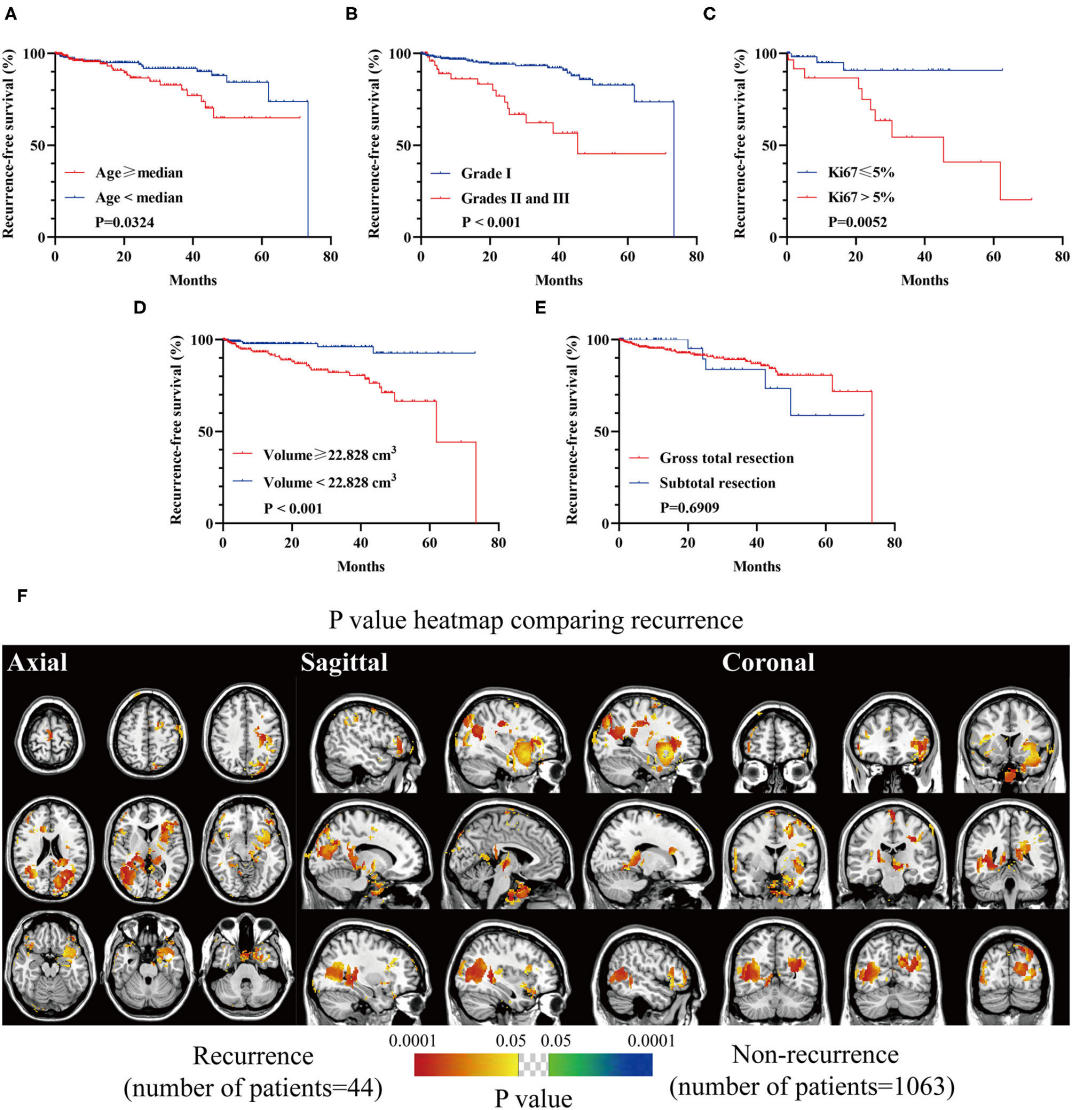
Recurrence-free survival (RFS) analyses of biological characteristics and *p*-value heatmap of meningioma comparing recurrence. **(A)** RFS analysis comparing older (age ≥ 56 years) with younger (age < 56 years) patients. **(B)** RFS analysis comparing WHO grade I lesions with grades II and III ones. **(C)** RFS analysis comparing Ki67 > 5% lesions with Ki67 ≤ 5% ones. **(D)** RFS analysis comparing larger preoperative lesions (≥22.828 cm^3^) with smaller ones (<22.828 cm^3^). **(E)** RFS analysis comparing gross total resection with subtotal resection. **(F)** *P*-value heatmap showed statistically significant clusters after comparing gross total resection with subtotal resection. The color ranging from green to dark blue, and bright yellow to red, both indicated the *p*-value from 0.05 to 0.0001.

Inferior RFS was observed in older patients (*p* = 0.0324, Figure 7A), high-grade lesions (*p* < 0.001, Figure 7B), Ki67 > 5% (*p* = 0.0052, Figure 7C), and a larger preoperative size (≥22.828 cm^3^, *p* = 0.0003, Figure 7D). The relation between EOR and RFS was not significant (Figure 7E). Clusters in occipital convexity, tentorium, sellar regions, parasagittal sinus, and left sphenoid wing were identified containing high a proportion of recurrent meningiomas (Figure 7F).

## Discussion

The present study visualized the preferred locations of meningioma in 1,107 patients according to different biological characteristics by voxel-wise constructing stereospecific frequency and *p*-value heatmaps, which could be a valuable reference for clinical decisions.

The high incidence rates and surgical difficulties for uneasily accessible locations of meningioma challenge the clinicians despite the benign nature of this tumor. As symptoms and treatment strategies largely depend on the location, it is indispensable to analyze the role of lesion location in clinical investigation of meningiomas (19, 20). A visual-perceivable graphic pattern is required for identifying and visualizing tumor locations rather than complicated table analyses. Voxel-based image normalization and segmentation are practical methods to comprehensively show the frequency of spatial distribution (15). The voxel-wise method was applied by Hirayama et al. (12) for meningioma mapping. The preferred locations of meningioma at the anterior two-thirds of the superior sagittal sinus and falx, olfactory groove, tuberculum sellae/planum sphenoidale/anterior clinoid process, parasellar/cavernous sinus, and cerebellopontine angle in our results were in accordance with the findings by Hirayama et al. The present study increased the sample size and objectively indicated the laterality of meningioma, overcoming their limitation of the intentional assumption that lesions distributed equally between hemispheres. The result that numerous lesions located at the middle third of the sagittal plane was reported in another study and was associated with a high risk of perioperative complications (21). The key points of surgery of these lesions were handling the feeding artery, protecting the central sulcus vein, and treating affected sagittal sinus (22). The distinct distribution in these areas could be explained by regionally thick arachnoid membranes with high risk of developing neoplasms. This theory was also verified by the large tumor size in the parasagittal sinus, tentorium, sphenoid wing, and olfactory groove by our results.

The Fisher's exact test was used for further exploration of the correlation between locations and biological characteristics of meningioma, for the significance calculation of voxels, and for the comparison of the two phenotypes. The method was proposed by Ellingson et al. (13, 14) for the laterality study of glioblastoma in terms of particular molecular and genetic profiles. In our study, age, sex, WHO grade, and extent of resection and recurrence were considered for comparison. Several interesting clusters were highlighted. The frontal and occipital structures were frequently associated with older and male patients and high-grade meningiomas, as previously described (23). A predominance in the right CPA was observed in the female sex, and lesions in this region were statistically prone to be WHO grade I. Although skull-base clusters also significantly indicated grade I lesions, they were identified as the most frequent occurrence in subtotal resection. It has been well-documented that skull-base meningioma was a risk factor for incomplete resection (24). Moreover, the p value heatmap suggested the skull-base lesions presented a significant inclination to recur. Therefore, more appropriate surgical approaches should be taken in skull-base lesions resection to decrease recurrence.

The meningiomas were histologically categorized into 15 subtypes, according to the 2016 WHO classification (2). The subtype is an important biological characteristic of meningiomas. However, over classification into 15 categories will decrease the statistical power. Another two reasons for not including the subtypes are the following: (1) the methodology of the voxel-wise analysis requires paired features (like WHO grade I vs. WHO grades II and III), and analyzing up to 15 categories simultaneously is difficult; and (2) some early pathological reports did not provide detailed subtype information. Future studies may focus on the different subtypes of meningiomas using other statistical methods.

As the molecular alterations might be responsible for the heterogeneity of meningioma, the expression of p53, Ki67, PR, EMA, and CD34 were further speculated to be related to the preferred locations of meningioma (23, 25). Expression of p53 is an indicator of the possible mutation of tumor suppressor gene *p53*, and Ki67 antigen protein is a cellular proliferative marker. The two markers predicted oncogenic ability and malignant degree (26, 27). EMA and PR were described as markers identifying a more advanced differentiated state (23). CD34 is commonly used for evaluating neovascularity and tumor behavior (23). Our results revealed that positive p53 and higher Ki67 lesions presented strong predominance in the falx, frontal and parietal convexity, and bilateral parasellar/cavernous sinus, which was consistent with the investigation by Maiuri et al. (17). This is probably associated with the distinct distribution of high-grade meningiomas. No significant cluster was noticed for PR, and meningioma with negative EMA predominantly located at the olfactory groove and middle fossa. However, Maiuri et al. pointed out that 75% of cases with PR expression >50% were located at the medial skull base (17). The high incidence of female patients was associated with the expression of PR in meningiomas. Furthermore, our results interestingly found that the proportion of male patients in the higher grades subgroup was significantly increased compared with that in the grade I subgroup, partially in accordance with the previous study (28). Higher CD34 levels were found in skull-base meningioma compared to the non-skull-base lesion by Haciyakupoglu et al. (29) and similar findings were found in sellar regions and sphenoid wing in our study.

According to the prognosis, there were expected findings that older patients, higher Ki67 expression, high-grade lesions, and a larger preoperative volume resulted in shorter RFS. Falcine and tentorial meningiomas were reported to have a high chance of recurrence, partially in agreement with our results (30). However, no superior RFS was shown in the gross total resection group. The role of the Simpson grading system for predicting recurrence was questioned, but Nanda et al. (6) maintained its prognostic value. It was reported that a higher risk of recurrence could be observed in STR for convexity lesions. In contrast, the recurrence was not correlated with the EOR for falx and posterior fossa lesions, leading to the unequally prognostic value of Simpson grading in terms of tumor locations (9). Further studies are needed to investigate the prognostic value of the Simpson grading system.

There are several limitations in the present study. First, the retrospective nature of the current study can be challenging. A prospective study, controlling field strength, imaging-section thickness, and applying fully automatic segmentation to improve accuracy, and analyzing more clinical characteristics [e.g., presenting symptoms, Karnofsky performance status (KPS), and postoperative radiotherapy] to improve prognostic prediction, is warranted. Notably, it is believed that obtaining a Simpson grade I resection for the skull-base meningiomas is difficult, and it may be inaccurate to evaluate the Simpson grade based on surgical reports or pre- and postoperative MRI. Thus, complete surgical video records might be more accurate for further evaluating the EOR of skull-base lesions in a retrospective study. Following that, the mechanism of preferred locations (e.g., laterality) of meningioma should be investigated in the future. Additionally, the genomic mutations were not analyzed in the present study. The clinical significance of the mutations of NF2, KLF4, and TRAF7 were proven in meningiomas, and studies have shown a significant association between mutations and specific locations (5, 31). The preferred locations of meningioma according to distinct mutations are to be analyzed in our following study. Lastly, it was not reported in the previous literature whether the voxel-wise Fisher's exact tests have multiple comparison problem when applied in multiple hypotheses testing. The implications of our research via Fisher's exact tests may give a hint to the nature of meningiomas but need further test by more prudent statistical analysis.

In conclusion, this is the first study visualizing the preferred locations of meningioma according to different biological characteristics by voxel-wise constructing stereospecific frequency and *p*-value heatmaps in a large surgery-treated patient cohort. Our findings might be a valuable reference for clinical decisions.

## Data Availability Statement

The data for this study can be acquired with appropriate request to the corresponding author.

## Ethics Statement

The inclusion process was approved by the ethical committee on human clinical research of the Second Affiliated Hospital of Zhejiang University. A general informed consent agreement, stating that the clinical, pathological, and imaging data with privacy protection might be used for teaching and scientific research, was signed by every patient as soon as hospitalized. Because of the retrospective nature of the current study with no clinical intervention, the specific informed consent agreement to a project was waived by the ethical committee. Medical records were desensitized for privacy protection.

## Author Contributions

CS and GC designed the study. JZ, QY, and XW collected the medical data. HZ, CZ, and YP made normalization and segmentation. ZD, JW, and FY analyzed data and constructed heatmaps. XY and JL performed survival and statistical analyses. CS and ZD wrote the manuscript. YI, BJ, QX, and GC reviewed all the data, results, and manuscript. All authors read and approved the final version of the manuscript. All authors contributed to the article and approved the submitted version.

## Conflict of Interest

The authors declare that the research was conducted in the absence of any commercial or financial relationships that could be construed as a potential conflict of interest.
