# Supplementary material for: The Preferred Locations of Meningioma According to Different Biological Characteristics Based on Voxel-Wise Analysis
Source: Front Oncol. 2020 Aug 21;10:1412. doi: 10.3389/fonc.2020.01412 (PMC7472960; doi:10.3389/fonc.2020.01412)
Supplement: Supplementary file 1 [file Data_Sheet_1.docx]

Supplementary Material


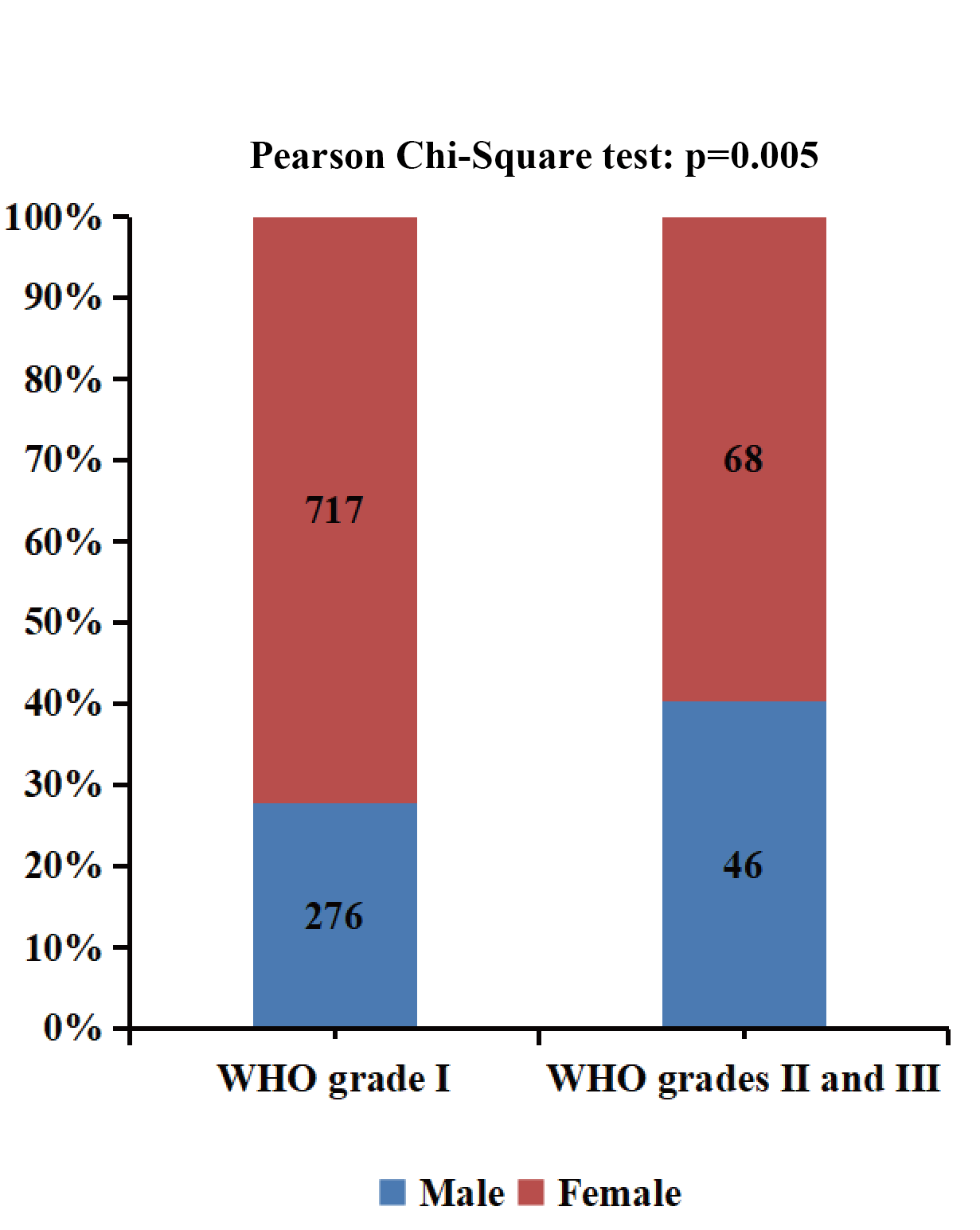


**Supplementary Figure 1.** Percentage stacked bar chart showed the gender distribution according to different WHO grades of meningiomas (Pearson Chi-Square test, p=0.005).


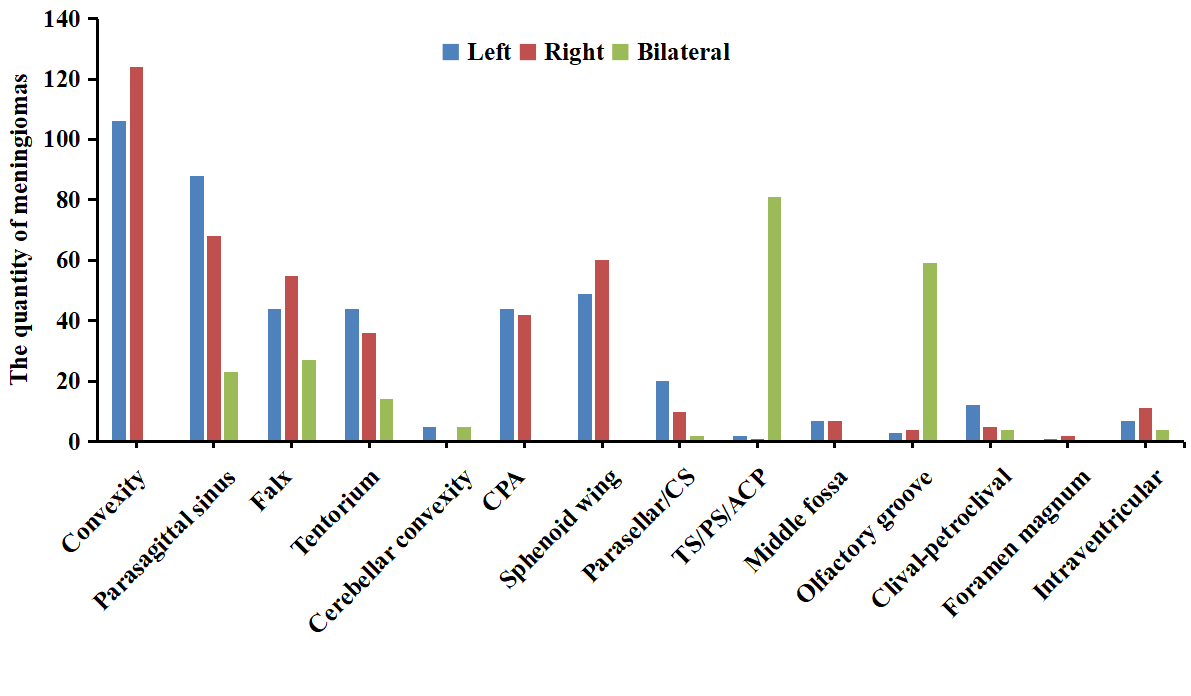


**Supplementary Figure 2.** The quantity of meningiomas in different locations and different sides probably showed the laterality. CPA refers to the cerebellopontine angle; CS refers to cavernous sinus; TS/PS/ACP refers to tuberculum sellae/planum sphenoidale/anterior clinoid process.

**Supplementary Table 1.** The side comparison of meningiomas in different biological characteristics.

|  | Left quantity | Right quantity | Pearson Chi-Square test (p value) |
| --- | --- | --- | --- |
| Age |  |  | 0.076 |
| Age ≥ median | 222 | 245 |  |
| Age < median | 220 | 191 |  |
| Sex |  |  | 0.959 |
| Male | 124 | 123 |  |
| Female | 318 | 313 |  |
| WHO grade |  |  | 0.160 |
| Grade I | 403 | 385 |  |
| Grades II and III | 39 | 51 |  |
